# A Combined DNA-Affinic Molecule and N-Mustard Alkylating Agent Has an Anti-Cancer Effect and Induces Autophagy in Oral Cancer Cells

**DOI:** 10.3390/ijms13033277

**Published:** 2012-03-09

**Authors:** Wen-Liang Lo, Pen-Yuan Chu, Tsung-Heng Lee, Tsann-Long Su, Yueh Chien, Yi-Wei Chen, Pin-I Huang, Ling-Ming Tseng, Pang-Hsien Tu, Shou-Yen Kao, Jeng-Fan Lo

**Affiliations:** 1 Division of Oral and Maxillofacial Surgery, Department of Stomatology, Taipei Veterans General Hospital, Taipei 11217, Taiwan; E-Mail: sykao@vghtpe.gov.tw; 2 Department of Dentistry, Institute of Oral Biology, School of Dentistry, National Yang-Ming University, Taipei 11217 Taiwan; E-Mail: polo661124@yahoo.com.tw; 3 Laryngology and Head and Neck Surgery, Department of Otolaryngology, Taipei Veterans General Hospital, Taipei 11217 Taiwan; E-Mail: pychu@vghtpe.gov.tw; 4 Institute of Biomedical Sciences, Academia Sinica, Taipei 115, Taiwan; E-Mails: tlsu@ibms.sinica.edu.tw (T.-L.S.); btu@ibms.sinica.edu.tw (P.-H.T.); 5 Institute of Clinical Medicine, National Yang-Ming University, Taipei 11217 Taiwan; E-Mails: g39005005@gmail.com (Y.C.); chenyw@vghtpe.gov.tw (Y.-W.C.); pinihuang@gmail.com (P.-I.H.); lmtseng@vghtpe.gov.tw (L.-M.T.); 6 Department of Medical Research and Education, Taipei Veterans General Hospital, Taipei 11217, Taiwan; 7 Cancer Center, Taipei Veterans General Hospital, Taipei 11217, Taiwan; 8 Department of Surgery, Taipei Veterans General Hospital, Taipei 11217, Taiwan

**Keywords:** BO-1051, AVO, autophagy, cell cycle, checkpoint kinases

## Abstract

Although surgery or the combination of chemotherapy and radiation are reported to improve the quality of life and reduce symptoms in patients with oral cancer, the prognosis of oral cancer remains generally poor. DNA alkylating agents, such as N-mustard, play an important role in cancer drug development. BO-1051 is a new 9-anilinoacridine N-mustard-derivative anti-cancer drug that can effectively target a variety of cancer cell lines and inhibit tumorigenesis *in vivo*. However, the underlying mechanism of BO-1051-mediated tumor suppression remains undetermined. In the present study, BO-1051 suppressed cell viability with a low IC_50_ in oral cancer cells, but not in normal gingival fibroblasts. Cell cycle analysis revealed that the tumor suppression by BO-1051 was accompanied by cell cycle arrest and downregulation of stemness genes. The enhanced conversion of LC3-I to LC3-II and the formation of acidic vesicular organelles indicated that BO-1501 induced autophagy. The expression of checkpoint kinases was upregulated as demonstrated with Western blot analysis, showing that BO-1051 could induce DNA damage and participate in DNA repair mechanisms. Furthermore, BO-1051 treatment alone exhibited a moderate tumor suppressive effect against xenograft tumor growth in immunocompromised mice. Importantly, the combination of BO-1051 and radiation led to a potent inhibition on xenograft tumorigenesis. Collectively, our findings demonstrated that BO-1051 exhibited a cytotoxic effect via cell cycle arrest and the induction of autophagy. Thus, the combination of BO-1051 and radiotherapy may be a feasible therapeutic strategy against oral cancer in the future.

## 1. Introduction

Oral cancer is the sixth most common cancer worldwide, and each year more than half a million cases are diagnosed [1]. Oral cancer is one of the most common types of head and neck cancer and is defined as malignant tumors occurring within the oral tissue including the lips, tongue, buccal mucosa, mouth floor, gums, oropharynx, hypopharynx, and hard palate. More than ninety percent of oral cancers are generated at the mucosal surface; these cancers are often referred to as oral squamous cell carcinoma. Sixty percent of these patients present with terminal stage oral cancer [2]. According to the statistics published by the Taiwan Department of Health in 2008, there was a significant increase in the incidence and mortality of oral cancer in Taiwan over the past decade. In Taiwan, chewing the betel nut, alcohol consumption, and smoking are the major risk factors for oral cancer. According to statistics published in 2008 by the Department of Health, malignant cancer ranks as the leading cause of death in Taiwan, and oral cancer has the sixth highest mortality rate. Although surgery or combination chemotherapy-radiation therapy can improve the quality of life and reduce symptoms in patients with oral cancer, 30 to 50% of patients survive less than three years.

Currently, the main clinical oral cancer treatments include surgery, radiation therapy, chemotherapy, and targeted drug treatment; however, oral cancer patients have high recurrence rates after surgery. Importantly, the relationships among the molecular mechanisms that induce cancer formation are complex. Thus, many pre-clinical trial drugs cannot fully destroy cancer cells due to molecular specificity and the relationship between drug dose and radiation resistance. Therefore, these drugs have not been approved for standard clinical use.

DNA alkylating agents play an important role in cancer drug development, with N-mustard being a representative agent. However, derivatives of N-mustard, such as melphalan and chlorambucil, have poor effects on cancer because they contain a nitrogen group (which is highly nucleophilic) and they lack a high degree of affinity to the DNA molecules [3]. Furthermore, these derivatives react easily with intracellular proteins or with small sulfur-containing molecules to generate glutathione [4], which is mutagenic to genes and toxic to the bone marrow; thus, these derivatives can increase the incidence of cancer [5]. Because the structure of N-mustards is not ideal for a candidate drug, scientists used N-mustard derivatives and DNA binding molecules such as acridines, anthraquinones, quinolines, phenanthridines, and 9-anilinoacridines to increase the chemical specificity of the drugs [6–8]. Su and Klionsky *et al*. designed and synthesized a series of N-mustard derivatives of 9-anilinoacridine based on the evidence mentioned previously. In that study, BO-1051 showed a remarkable ability to target a variety of cancer cell lines, including two drug-resistant cell lines, with lower half-maximal inhibitory concentration (IC_50_) than that of normal tissue [9]. In *in vivo* experiments, BO-1051 had a potent antitumor efficacy in nude mice bearing human breast MX-1 xenografts. BO-1051 also effectively suppressed human glioma U87MG xenografts in nude mice [9]. However, the underlying mechanism of cell death induced by BO-1051 was not determined.

Clinical anti-cancer drugs target specific molecular characteristics of cancer cells. As the growth rate of cancer cells is higher than that of normal tissue, high levels of DNA damage lead to cell cycle arrest and the initiation of cell death. Previous studies have indicated that BO-1051, as a DNA alkylating agent, can break double-stranded DNA and damage cancer cells, ultimately leading to their death [9]. In this study, the cytotoxic effects and mechanism of death induction of the anti-cancer drug BO-1051 were investigated in oral cancer cells. In addition, we assessed whether BO-1051 possessed tumor suppressive effects *in vivo* and whether the combination of BO-1051 with cobalt-60 radiation had an additive tumor suppressive effect on xenograft tumorigenesis in immunocompromised mice.

## 2. Results and Discussion

### 2.1. Cytotoxicity of BO-1051 in Oral Cancer Cell Lines

**Figure 1:**
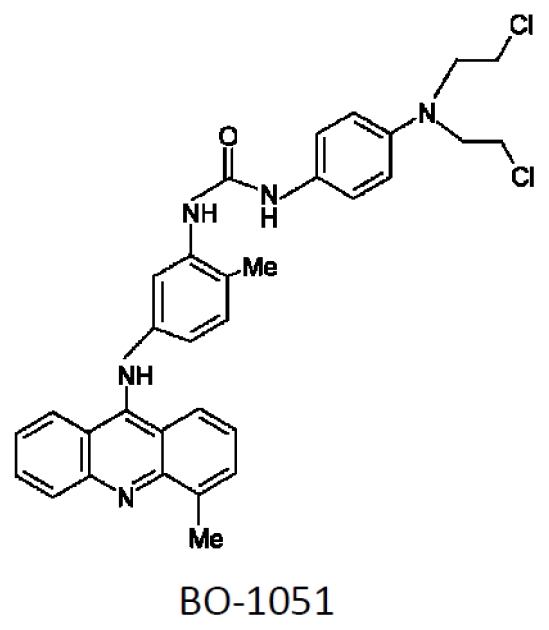
The chemical structure of BO-1051. The structural formula of 9-anilinoacridine N-mustard-derivative anti-cancer drug BO-1051.

**Figure 2:**
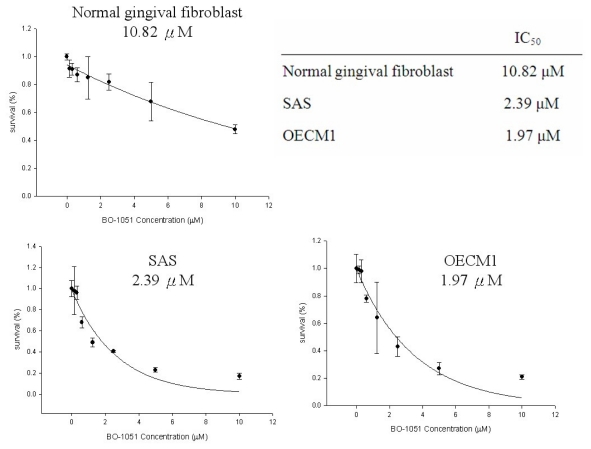
The dose response curve of BO-1051-induced cytotoxicity and determination of the IC_50_ value in normal gingival fibroblasts, SAS cells, and OECM1 cells. BO-1051 was sequentially diluted by the addition of DMSO and then added to the cells and cultured for 48 h. A MTT assay was used to analyze the viability and to determine the IC_50_ value in the oral cancer cell lines SAS and OECM1, as well as normal gingival fibroblasts, in response to BO-1051 treatment. The IC_50_ value of SAS cells, OECM1 cells, and normal gingival fibroblasts were 2.39 μM, 1.97 μM, and 10.82 μM, respectively.

The structure of BO-1051 is shown in Figure 1. To determine the toxic effects of BO-1051 on oral cancer cells, BO-1051 was sequentially diluted with DMSO and then added to the cells and cultured for 48 h. A MTT assay was used to analyze the viability of the OECM1 and SAS oral cancer cell lines, as well as normal gingival fibroblasts, in response to BO-1051 treatment. As shown in Figure 2, BO-1051 decreased cell viability slightly in normal gingival fibroblasts with an IC_50_ of 10.82 μM. In OECM1 and SAS, BO-1051 decreased cell viability robustly with IC_50_ values of 2.76 μM and 1.97 μM, respectively. Notably, the growth inhibition concentration of BO-1051 for the two oral cancer cell lines was significantly lower than that for the normal gingival fibroblasts, as reported previously [9]. Based on these results, we studied the dose effect of BO-1051 (including 0 μM, 0.1 μM, 0.5 μM, 1.0 μM, 1.5 μM, 2.5 μM, 5 μM, and 10 μM) in subsequent experiments and investigated the underlying mechanisms of BO-1051-mediated cytotoxicity in oral cancer cells.

### 2.2. Diverse Effect of BO-1051 on Cell Cycle in Normal Gingival Fibroblasts and Oral Cancer Cells

**Figure 3:**
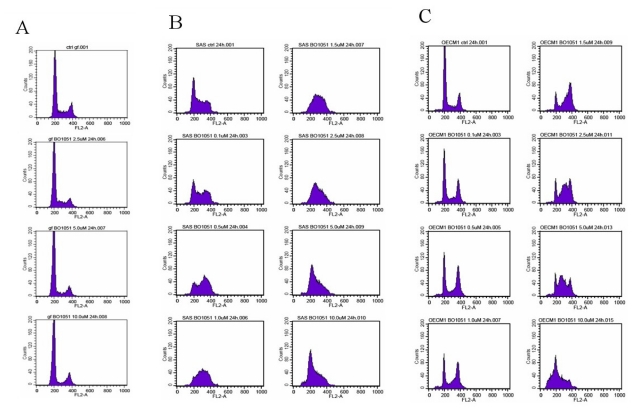
The effect of BO-1051 on the cell cycle profile of normal gingival fibroblasts, SAS cells, and OECM1 cells. (**A**) Normal gingival fibroblasts; (**B**) SAS cells or (**C**) OECM1 cells were exposed to increasing doses of BO-1051 (0 to 10 μM) for 24 h before collection and FACS analysis of the propidium iodide-stained cells was performed. Notice that BO-1051 induced G2/M cell cycle arrest in oral cancer cells (SAS cells and OCEM1 cells) but showed no effect on normal gingival fibroblasts.

To investigate the BO-1051-mediated cytotoxic effect on oral cancer cells further, we evaluated the effect of BO-1051 on the growth of oral cancer cells by conducting cell cycle analysis. In normal gingival fibroblasts, BO-1051 did not show an observable effect on cell cycle progression (Figure 3A). In both SAS and OECM1 cells (Figure 3B,C), correlating with increasing doses of BO-1051, the cell number in G0/G1 phase gradually reduced, while the cell number in S and G2/M phases increased, indicating a G2/M cell cycle arrest. At doses higher than 2.5 μM, the G2/M cell cycle arrest was further enhanced, and S phase cell cycle arrest was induced. There was no increase of cells in the sub-G1 phase with the different doses of BO-1051 (Figure 3). These data suggested that BO-1051 has an inhibitory role on oral cancer cells partially via the induction of cell cycle arrest.

### 2.3. BO-1051 Induced Autophagy in Oral Cancer Cells

**Figure 4:**
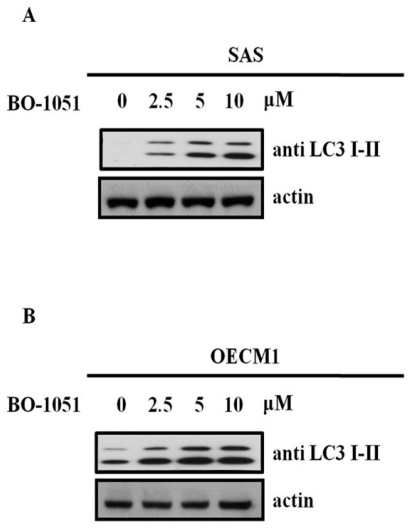
The effect of BO-1051 on LC3 I-II protein expression in (**A**) SAS and (**B**) OECM1 cells. The cultures were exposed to BO-1051 for 24 h, and the proteins extracted from harvested cells were resolved using SDS-PAGE, transferred to nitrocellulose and probed with LC3-I-II-specific antibodies. In response to increasing doses of BO1051 in these two oral cancer cell lines, LC3 I expression remained unchanged and LC3 II expression was increased in a dose-dependent manner.

**Figure 5:**
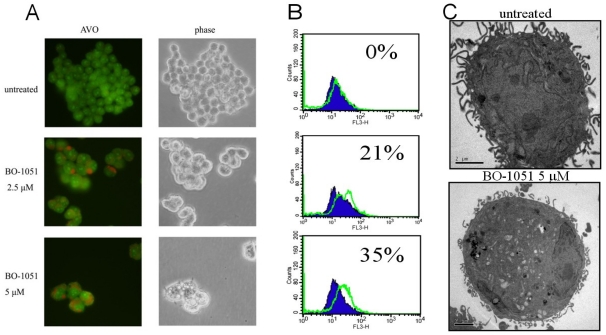
BO-1051 induces formation of acidic vesicular organelles (AVOs). (**A**) Induction of AVOs by BO-1051 in SAS cells. The cells were treated with various concentrations of BO-1051 and stained with acridine orange. Red fluorescence indicates the induction of AVOs; (**B**) Quantification of acridine orange staining was performed with flow cytometry; (**C**) Electron microscopy confirmed the induction of AVOs.

To determine if autophagy was involved in the BO-1051-mediated cytotoxicity, we measured the changes in the expression pattern of a known reliable marker of autophagosomes termed LC3, a mammalian homolog of yeast Atg8. Tracking the conversion of LC3-I to LC3-II is indicative of autophagic activity. After 48 h of BO-1051 treatment in both SAS and OECM1 cells, the LC3-I expression remained unaltered at all given doses of BO-1051, whereas BO-1051 led to a dose-dependent increase in LC3-II, especially at doses higher than the IC_50_ (Figure 4). These results suggested that BO-1501 induced autophagy in both oral cancer cell lines. The development of acidic vesicular organelles (AVOs) has been reported to be an additional characteristic of autophagy. Therefore, we evaluated the formation of AVOs using acridine orange staining in SAS cells. There was an increase in red fluorescence in SAS cells after BO-1051 treatment (Figure 5A, left panel). Subsequently, we quantified the staining using flow cytometry. BO-1051 treatment increased red fluorescence (Y-axis) in SAS cells, indicating the formation of AVOs was induced by BO-1051 (Figure 5B). Parallel investigations with electron microscopy further confirmed the BO-1051-induced formation of AVOs (Figure 5C).

### 2.4. BO-1051 Induced Checkpoint Kinase Phosphorylation

**Figure 6:**
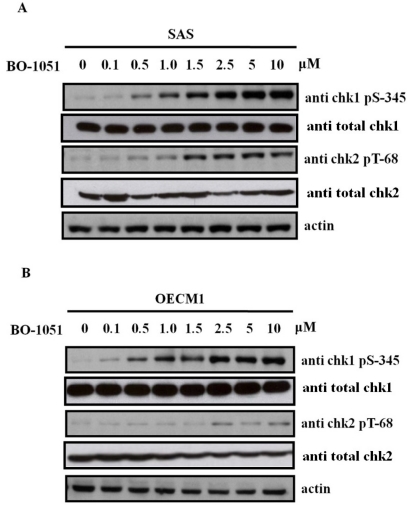
The effect of BO-1051 on chk1 serine-345 and chk2 threonine-68 protein phosphorylation. Cultures were exposed to BO-1051 for 24 h and the proteins extracted from harvested cells were resolved using SDS-PAGE, transferred to nitrocellulose and probed with Chk1- and Chk2-specific antibodies. In response to increasing doses of BO1051 in SAS cells and OCEM1 cells, Chk1 serine-345 and Chk2 threonine-68 phosphorylation was increased in a dose-dependent manner.

**Figure 7:**
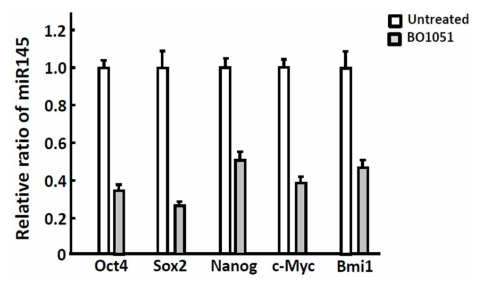
The effect of BO-1051 on the gene expression of stemness markers. Quantitative RT-PCR analysis of Oct4, Sox, Nanog, c-Myc and the oncogene Bmi-1 in the oral cancer cells with or without BO-1051 treatment was performed.

Cell cycle checkpoints are regulatory pathways that control the order and timing of cell cycle transitions and ensure that critical events such as DNA replication are completed with high fidelity [10]. When DNA damage occurs, the cell cycle arrest and DNA repair checkpoints are activated. The proteins ataxia telangiectasia-mutated (ATM) and ataxia-telangiectasia and Rad3-related (ATR) activate downstream checkpoint kinase 2 (chk2) and checkpoint kinase 1 (chk1), respectively, that undertake the repair of DNA double-strand breaks. As shown in Figure 6, BO-1051 induces the phosphorylation of both chk1 serine-345 and chk2 threonine-68 in dose-dependent manners, in both SAS and OECM1 oral cancer cells. These findings indicated that BO-1051-induced autophagy involves activation of the ATM/Chk2 and ATR/Chk1 pathways. A previous study has demonstrated that overexpression of stemness markers (e.g., Oct4 and Nanog) correlates with cisplatin resistance and cancer stem-like properties in oral squamous cell carcinoma [11]. Quantitative RT-PCR showed that BO-1051 treatment decreased the expression of stemness markers including Oct4, Sox2, Nanog and c-Myc and the oncogene Bmi-1, suggesting that BO-1051 suppressed the cancer stem-like and chemoresistant properties in oral cancers (Figure 7).

### 2.5. Tumor Suppressive Effects of BO-1051 on the Xenograft Tumors in Mice

**Figure 8:**
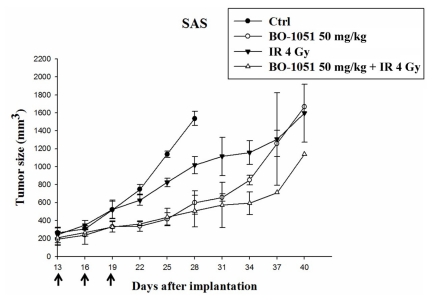
Effect of BO-1051 and/or radiation on xenograft tumors in mice. Nude mice were subcutaneously transplanted with 5 × 10^5^ SAS cells and randomized into 4 groups: control, radiation (4 Gy), BO-1051 (50 mg/kg) or BO-1051 plus radiation (50 mg/kg and 4 Gy, respectively). Thirteen days after implantation, the mice were intraperitoneally treated with BO-1051 once every two days for three courses of treatment (as indicated by arrow on days 13, 16 and 19 after implantation). Radiation (4 Gy) was delivered 24 h after the second injection of BO-1051. Note that BO-1051 alone extended the time course required for the tumor growth, indicating that this drug suppressed tumorigenesis *in vivo*. The combination of BO-1051 plus radiation further suppressed the progression of tumor growth.

To evaluate the tumor suppressive effects of BO-1051 *in vivo*, we performed a xenograft tumor assay in nude mice. Xenotransplantation of the oral cancer cell line SAS led to significant tumor formation in recipients, and the recipients with tumor volumes greater than 150 mm^3^ were randomized into the following four groups: control group; BO-1051 group (50 mg/kg b. w., given at intervals of 2 to 3 days); irradiation group (receiving 4 Gy radiation); and the combination of BO-1051 and irradiation group (50 mg/kg BO-1051 treatment as described above with 4 Gy radiation). The tumor volume of the control group increased from the average volume 200–250 mm^3^ to 1000 mm^3^ in approximately 11 days. The tumor volume of the BO-1051 group increased to a comparable volume at 22 days, the irradiation group in 14 days, and the BO-1051 plus radiation in 26 days. The longer time course required for the tumor growth in the BO-1051 plus radiation group demonstrated that BO-1051 moderately suppressed tumorigenesis *in vivo* and the combination of BO-1051 and radiation potentially suppressed the progression of xenograft tumor growth in mice (Figure 8).

### 2.6. Discussion

BO-1051 is a new DNA-affinic molecule and an N-mustard alkylating agent that is used as an anti-cancer drug. BO-1051 was created by combining two clinical cancer drugs, N-mustard and 9-anilinoacridine. Bifunctional alkylating agents induce collapsed replication forks that can lead to cell cycle arrest, DNA repair, or apoptosis [12]. Bifunctional N-mustard alkylating agents, such as BO-1051, are able to induce marked dose-dependent levels of DNA interstrand cross-linking, resulting in a broad spectrum of anti-cancer activities *in vitro* [13,14]. BO-1051 has been shown to possess therapeutic efficacy in nude mice bearing human breast MX-1 tumors and human glioma *in vivo* [9]. In recent years, the combination of radiation therapy with anti-cancer drugs has also advanced for the clinical treatment of cancer. Cisplatin, which has a mechanism of action similar to that of the N-mustards, is used in the clinical treatment of oral cancer patients and is often combined with radiation treatment. In the present study, we investigated the synergistic cytotoxic effects of BO-1051 and radiation on oral cancer cells and tumor xenografts.

The mechanism of the BO-1051-induced cytotoxic effect has been reported in glioma cells [15] and hepatocellular carcinoma cells [16]. BO-1051 has been shown to inhibit the growth of glioma cells, which are notorious for the high resistance to radiotherapy. Our recent study further demonstrated that BO-1051 significantly enhanced the radiosensitivity of tumor cells. The enhanced radiosensitivity was found to be associated with a G2/M phase arrest as well as sustained DNA damage. *In vivo* studies further demonstrated that BO-1051 enhanced the radiotherapeutic effects on glioma-bearing xenograft tumors [15]. In hepatocellular carcinoma cell lines, BO-1051 simultaneously induced apoptosis and autophagy. DNA double strand breaks induced by BO-1051 activated the ATM signaling pathway and subsequently resulted in caspase-dependent apoptosis [16]. In the present study using oral cancer cell lines, G2/M phase arrest, induction of autophagy, as well as radiosensitization was observed, which was similar to our previous findings [15]. We further demonstrated that BO-1051 decreased the expression of stemness markers, suggesting that such potential chemotherapeutic drug also suppressed cancer stem-like and chemoresistant properties. However, there was no obvious evidence showing that BO-1051 led to apoptosis in such cells. These findings indicated that the mechanisms mediating the BO-1051-induced cytotoxicity could be cell-specific.

In this study, the induction of autophagy was demonstrated by the conversion of LC3-I to LC3-II, with the flow cytometry data and electron microscopic evidence showing the development of AVOs. Nevertheless, the role of autophagy has been contested by numerous studies because autophagy has been reported to be either prodeath or prosurvival [17]. In hepatocellular carcinoma cell lines, autophagy can be induced by various compounds and can be involved in cell death or cytoprotection [18–20]. Inhibition of autophagy at different stages has been reported to have opposite effects on cell survival [21]. Our previous findings in hepatocellular carcinoma also showed that inhibition of autophagy leads to enhanced apoptosis in both early and late stages, indicating that autophagy is not strictly a prodeath mechanism [16]. However, in glioma [22,23] and lung cancers [24], there were several lines of evidence supporting autophagy as one of the causes of radiosensitization instead of apoptosis, which was compatible with our findings of BO-1051-induced radiosensitization in oral cancer cells in the present study. Therefore, the correlation between cytotoxicity, autophagy, and radiosensitivity induced by BO-1051 needs to be further investigated. In future investigations, it will be necessary to elucidate the differential damage, such as the interstrand DNA crosslinking in normal and cancer cells, the bone marrow and hematological toxicity *in vivo*, and the resistance determinants such as the glutathione and glutathione S-transferase levels. Studies like these may assist to determine the feasibility and potential of this DNA-reactive compound for clinical use.

## 3. Materials and Methods

### 3.1. MTT Assay

The cells were seeded in 96-well (6000 cells per well) or 24-well (30,000 cells per well) plates in complete culture medium. After overnight culture, the medium was replaced with either solvent or chemicals at indicated concentrations in complete medium. The cells were cultured until the time indicated, and an MTT assay was performed. In brief, cells were stained with 0.1 mg/mL MTT (Sigma) for 2–4 h and then dissolved in DMSO (Sigma). MTT values were measured at 570 nm using a microplate reader.

### 3.2. Cell-Cycle Analysis

After treatment, the cells were prepared for fluorescence-activated cell sorting (FACS) to assess the relative distribution in the respective phases of the cell cycle. Cells were harvested 24 h after treatment with BO-1051, pelleted using centrifugation, resuspended in PBS, fixed in 70% ethanol and stored at −20 °C. Immediately before flow cytometric analysis, the cells were washed in cold PBS (4 °C), incubated in Ribonuclease A (Sigma) for 20 min at room temperature, labeled by adding an equal volume of propidium iodide solution (100 μg/mL) and incubated in the dark for 20 min at 4 °C. These samples were measured (20,000 events collected from each) in a FACSCalibur cytometer (BD FACS Caliber; Mountain View, CA, USA). The data shown are for one experiment, but the results were reproduced and confirmed in at least three identical experiments.

### 3.3. Immunoblot Analysis

Harvested cells were pelleted using centrifugation, washed with PBS, and lysed with RIPA buffer. Protein content was measured with a protein assay kit (Bio-Rad Laboratories, Hercules, CA, USA). Fifty micrograms of total protein were separated by SDS/PAGE (10% or 12% gels) and transferred to nitrocellulose membranes (Pall Corporation, Ann Arbor, MI, USA) for immunological detection of proteins. The blots were probed using antibodies against cleaved PARP, cleaved caspase-3, and beta-actin (Millipore Corporation, Milford, MA, USA).

### 3.4. *In Vivo* Tumorigenesis Model

Six-week-old female nude mice were used in these studies. Mice were caged in groups of five or less, and all animals were fed a diet of animal chow and water *ad libitum*. All procedures involving animals were performed in accordance with the institutional animal welfare guidelines of the Taipei Veterans General Hospital. Tumors were generated by injecting 5 × 10^6^ SAS cells subcutaneous (s.c.) into the right hind leg. Irradiation was performed using a T-1000 Theratronic cobalt unit (Theratronic International, Inc.; Ottawa, Canada) irradiator with animals restrained in a custom jig.

### 3.5. Statistical Analysis

Data were expressed as mean ± SD from at least three independent experiments. Significant differences between groups were detected by using the AVONA or unpaired Student’s *t* tests. The results were considered statistically significant at *p* < 0.05.

## 4. Conclusions

Oral cancer is the sixth most common cancer worldwide and usually is accompanied with an extremely poor prognosis. Our findings demonstrated that BO-1051 inhibited the growth of oral cancer cells with G2/M phase arrest, downregulation of stemness markers and upregulation of checkpoint kinases, indicating that BO-1051 can induce DNA damage and participate in DNA repair mechanisms. Our *in vitro* findings also demonstrated that BO-1051-induced autophagy, which is highly associated with radiosensitization in other cell lines. Furthermore, *in vivo* studies demonstrated that BO-1051 enhanced the radiotherapeutic effects on SAS xenograft tumors in immunocompromised mice. In this model, the combination of BO-1051 plus radiation produced the best therapeutic response. These data suggest that BO-1051 provides a new strategy to improve therapeutic efficacy by radiation therapy.
